# Endoscopic versus catheter-based minimally invasive evacuation of spontaneous supratentorial intracerebral hemorrhage: a systematic review and meta-analysis

**DOI:** 10.1007/s10143-026-04426-3

**Published:** 2026-08-05

**Authors:** Shaan Patel, Shiva A. Nischal, Kush M. Kale, Angelette Mendonca, Keenan Piper, Pious D. Patel, Michael R. Gooch, Stavropoula I. Tjoumakaris, Pascal M. Jabbour

**Affiliations:** 1 Department of Neurological Surgery, Thomas Jefferson University Hospital, Philadelphia, PA 19107 USA; 2 Department of Physiology, Anatomy & Genetics, Medical Sciences Division, University of Oxford, Oxford, OX1 3PT UK; 3 School of Clinical Medicine, University of Cambridge, Cambridge, CB2 0SP UK

**Keywords:** Minimally invasive, Intracerebral hemorrhage, Endoscopic, Catheter

## Abstract

Minimally invasive surgery (MIS) for spontaneous supratentorial intracerebral hemorrhage (sICH) includes endoscopic evacuation and catheter-based evacuation (aspiration or drainage ± thrombolysis), but comparative performance remains uncertain. We performed a systematic review and meta-analysis comparing MIS approaches. PubMed, Embase, and CENTRAL were searched (December 2025) for studies in adults undergoing endoscopic or catheter-based MIS for supratentorial sICH. Random-effects meta-analysis was conducted. Prespecified subgroup analyses stratified catheter-based techniques by thrombolysis use. Risk of bias and certainty of evidence was assessed. Twenty-nine studies (8221 patients; 5041 endoscopic; 3180 catheter-based) were included. Endoscopic evacuation was associated with lower mortality (RR 0.64; 95% CI: 0.47–0.87; *P* < 0.01; *I*^2^ = 44.8%), rebleeding (RR 0.58; 95% CI: 0.39–0.87; *P* < 0.01; *I*^2^ = 22.2%), postoperative hematoma volume (MD -5.6 mL; 95% CI: -10.30–0.90; *P* < 0.05; *I*^2^ = 96.9%), and higher day-one evacuation rate (MD 24.5%; 95% CI: 14.60–34.40; *P* < 0.001; *I*^2^ = 99.7%). Overall complications (RR 0.98; 95% CI: 0.78–1.25; *P* = 0.90; *I*^2^ = 48.5%) and infection (RR 1.03; 95% CI: 0.64–1.66; *P* = 0.05; *I*^2^ = 43.6%) were similar. Catheter-based approaches had lower intraoperative blood loss (MD 69.0 mL; 95% CI: 27.7–110.4; *P* < 0.001; *I*^2^ = 96.9%). Subgroup analyses suggested that mortality and evacuation rate differences favored catheter-based cohorts receiving thrombolysis. Functional outcomes diverged: GCS/GOS favored endoscopy (SMD 0.27) while mRS marginally favored catheter-based approaches (MD 0.28), a between-group difference below the threshold generally considered clinically meaningful. Endoscopic evacuation was associated with lower mortality, rebleeding, and residual hematoma burden, while catheter-based approaches retained perioperative efficiency advantages. Functional findings were mixed and of uncertain clinical significance. Given predominantly non-randomized evidence and heterogeneity, these findings should be interpreted as strategy-specific associations.

**Supplementary Information:**

The online version contains supplementary material available at 10.1007/s10143-026-04426-3.

## Introduction

Spontaneous intracerebral hemorrhage (sICH) is a lethal, disabling stroke subtype, comprising 10–20% of strokes and carrying ~ 40% one-month mortality despite modern neurocritical care [2, 14]. Among survivors, outcome reflects not only hematoma volume and location but also secondary injury: mass effect, perihematomal edema (PHE), microvascular compromise, and neuroinflammation that evolves over hours to days and may be mitigated if clot burden is reduced early [25, 56]. Yet surgery is not simply “remove the blood.” Open craniotomy requires tissue disruption, risks iatrogenic injury, and randomized trials have not shown superiority over best medical management, underscoring a central tension: therapeutic efficacy must be achieved without exchanging hematoma-related injury for iatrogenic harm [41].

Minimally invasive surgery (MIS) aims to resolve this trade-off by reducing clot burden through smaller corridors, differing in access geometry, evacuation kinetics, and hemostatic control [29]. Endoscopic evacuation (including SCUBA) provides direct visualization for debulking and enables identification and control of active bleeding points [26]. Catheter-based drainage with adjunctive thrombolysis (including ADIWT, as operationalized in MISTIE protocols) prioritizes stereotactic accuracy and minimal parenchymal disruption, achieving evacuation gradually through catheter drainage and clot lysis [19]. Stereotactic craniopuncture aspiration (including CRANIO-CAN) aims for rapid volume reduction via targeted aspiration, typically without prolonged thrombolytic dwell[29]. The latter two are grouped as catheter-based MIS – an umbrella term for the non-endoscopic stereotactic family, encompassing catheter drainage (± thrombolysis) and stereotactic aspiration via cannula, syringe, or puncture needle – reflecting shared dependence on target accuracy and liquefaction kinetics [29]. We retain “catheter-based” for brecity, recognizing that an indwelling catheter was not used in every comparator study.

These strategies encode distinct mechanistic trade-offs. Endoscopy offers intuitive advantages in visual control, cavity inspection, and immediate hemostatic intervention, which may translate into better clot removal. Refinements, such as dry-field followed by wet-field evacuation, preserve early visualization and facilitate cavity clearance [26]. Catheter-based approaches, by contrast, trade visualization for procedural minimalism and tissue-sparing access. However, reliance on delayed clot dissolution introduces vulnerabilities, including catheter obstruction, incomplete evacuation, and infection or thrombolysis-associated rebleeding [19]. Importantly, because catheter-based evacuation is time-dependent, its effectiveness is coupled to hematoma consistency and the ability to achieve a threshold of volume reduction -factors that may explain why benefits appear sensitive to patient selection and performance [19, 49].

The clinical evidence base reflects these mechanistic tensions. Trials of tissue plasminogen activator (tPA)-assisted catheter drainage have not demonstrated consistent functional superiority over standard care, although post-hoc analyses suggest that patients achieving greater volume reduction may derive benefit [49]. Comparative observational studies suggest that endoscopic approaches may deliver more complete and immediate evacuation than catheter-based strategies, with downstream consequences for mass effect and PHE evolution [32]. However, most available trials compare individual MIS techniques against conservative management or open surgery rather than directly against each other. As a result, apparent differences between strategies may reflect confounding by indication, timing, hematoma phenotype, or outcome definition rather than true technique-specific effects.

Accordingly, the key uncertainty is which MIS strategy optimally balances evacuation efficiency and safety for a given supratentorial sICH phenotype. We performed a systematic review and meta-analysis synthesizing comparative evidence across MIS strategies, evaluating efficacy and safety, with prespecified subgroup analyses comparing endoscopic versus catheter-based techniques, including stratification by thrombolytic use.

## Materials and methods

### Study design and registration

This systematic review and meta-analysis was conducted in accordance with the PRISMA guidelines [47] and adhered to AMSTAR-2. The study protocol was registered a priori on PROSPERO (CRD420251270625).

### Search strategy

PubMed, Embase, and CENTRAL were searched from database inception to 10th December 2025. Search terms combined controlled vocabulary and free-text keywords relating to sICH, MIS and OS approaches (Supplementary Table 1) and were restricted to English-language human studies. Reference lists of included studies and relevant prior reviews were screened manually.

### Eligibility criteria

Inclusion criteria were: (i) randomized controlled trials (RCTs) or comparative observational studies; (ii) adults (≥ 18 years) with supratentorial sICH; (iii) endoscopic (dry-field (air-only) or mixed fluid-air techniques) or catheter-based MIS, defined here to ecnompass catheter drainage ± thrombolysis and stereotactic craniopuncture aspiration via cannulla, syringe, or puncture needle; and (iv) reporting at least one operative or clinical endpoint of interest.

Exclusion criteria were infratentorial ICH, non-parenchymal hemorrhage, isolated intraventricular hemorrhage without parenchymal involvement, small case series (< 5 patients), conference abstracts, cadaveric or technical reports, reviews, and studies without extractable comparative data.

### Outcomes

Primary outcomes were: (i) overall complications (composite of reported perioperative or postoperative adverse events); (ii) evacuation rate, defined as ((preoperative hematoma volume—postoperative hematoma volume)/preoperative hematoma volume, %), (iii) rebleeding; (iv) infection; (v) all-cause mortality. Secondary outcomes included intensive care unit (ICU) and hospital length of stay (LOS), neurological and functional outcomes (Glasgow Coma Scale (GCS), Glasgow Outcome Score (GOS), Modified Rankin Scale (mRS), intraoperative blood loss, operative time, and postoperative residual hematoma volume.

### Study selection

Two reviewers independently screened titles and abstracts using Rayyan followed by full-text assessment. Disagreements were resolved by consensus with senior adjudication when required. Inter-rater reliability was high (*κ* = 0.86).

### Data extraction

Data was extracted using a prespecified template capturing study characteristics, patient demographics, hemorrhagic characteristics, operative details (MIS subtype and technical approach), and outcomes of interest. For the composite overall complications endpoint, we recorded study-defined perioperative and postoperative adverse events, noting that minor self-limiting events were inconsistently reported and could not be uniformly separated across studies.

### Risk of bias, and certainty of evidence

Risk of bias was assessed using Cochrane’s Risk-of-Bias tool 2 (RoB 2) [53] for randomized trials and ROBINS-I [52] for non-randomized studies. Certainty of evidence for each outcome was evaluated using GRADE [50].

### Statistical analysis

Random-effects meta-analysis using restricted maximum-likelihood estimation was used to account for heterogeneity. Dichotomous outcomes were summarized as risk ratios (RRs) and continuous outcomes as mean differences (MDs) with 95% confidence intervals (CIs). Significance level was set at *α* < 0.05. Leave-one-out sensitivity analyses were conducted for statistically significant outcomes. To assess whether the between-arm baseline imbalance influenced the efficacy findings (mortality, rebleeding, and day-one evacuation rate), we performed random-effects meta-regression of each study-level effect on the between-arm difference (endoscopic minus catheter-based) in baseline hematoma volume and in preoperative GCS, modeling one covariate at a time with Knapp-Hartung variance, and conducted subgroup analyses by study design (randomized versus non-randomized) and geographic region. Publication bias was assessed using funnel plots and Egger’s regression. All analyses were performed using R v4.3.2 (*meta* package [3]).

## Results

**Fig. 1:**
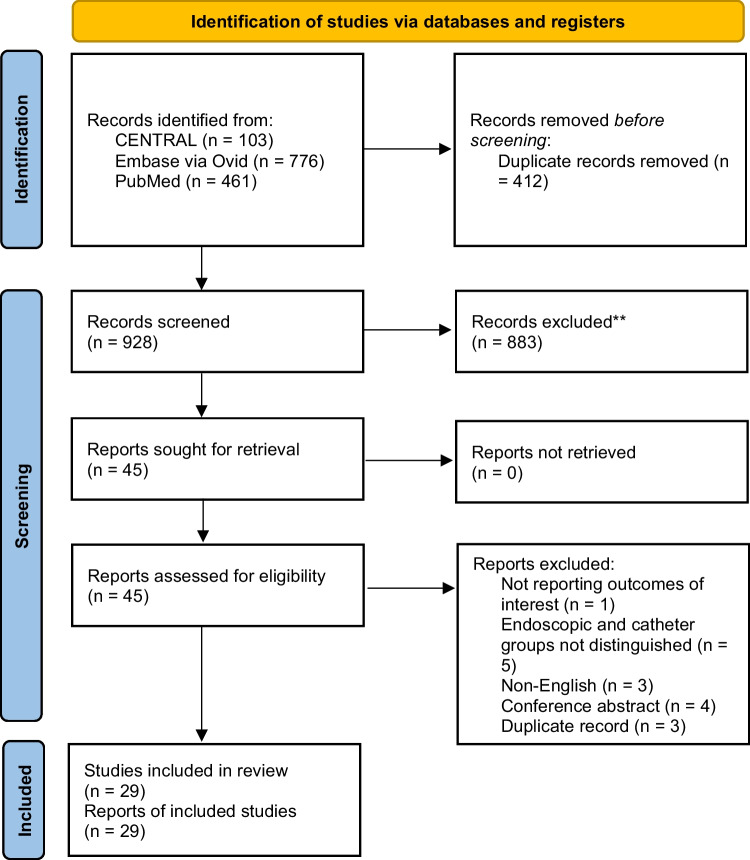
PRISMA flow diagram of study selection. Preferred Reporting Items for Systematic Reviews and Meta-Analyses (PRISMA) flow diagram outlining the study selection process. The number of records identified, screened, assessed for eligibility, and included in the final analysis are detailed at each stage

A total of 1340 records were identified, of which 29 studies [4–8, 10, 11, 13, 15–17, 20, 24, 28, 31–33, 35, 40, 44, 45, 51, 54, 55, 57–61] met the inclusion criteria, comprising 8221 patients (5041 endoscopic; 3180 catheter-based) (Fig. 1). Sample sizes ranged from 8 to 3032 participants. There were 4 RCTs, 24 retrospective cohorts, and one prospective cohort study. Most studies were conducted in East Asia (China *n* = 25; Japan *n* = 2; South Korea *n* = 1), with one study in the United States. Nineteen studies compared endoscopic evacuation with stereotactic catheter-based drainage using adjunctive thrombolysis (tPA), while ten studies compared endoscopic evacuation with stereotactic aspiration without thrombolysis. Mean age was 59.97 years in endoscopic cohorts and 60.55 years in catheter-based cohorts with a mean follow-up of 5.9 months. Baseline characteristics are summarized in Table 1, and surgical characteristics in Supplementary Table 2. Compared with catheter-based cohorts, endoscopic cohorts had slightly larger baseline hematoma volumes (45.04 versus 41.43 mL; MD 1.81; 95% CI: 0.40–3.23; *P* < 0.05) and marginally lower pre-operative GCS (8.9 versus 9.1; MD −0.28; 95% CI: −0.55–−0.01; *P* < 0.05), while time to surgery did not differ significantly (15.8 versus 18.9 h; MD −2.45; 95% CI: −7.19–2.30; *P* = 0.31) (Supplementary Table 3). Given these baseline differences, the subsequent comparative estimates should be interpreted with caution and are addressed in sensitivity analyses below. The present review met high methodological quality by AMSTAR-2 (Supplementary Table 4).

**Table 1:** Baseline characteristics of included studies

Author,year	Country	Study design	Sample size		Age (years) (mean (SD) or mean (range))		Sex (% female)		Comorbidities		ICH location		IVH bleed (n)		Hematoma volume (mL)		Preoperative GCS		Time to surgery (hours)	
			ES	CA	ES	CA	ES	CA	ES	CA	ES	CA	ES	CA	ES	CA	ES	CA	ES	CA
Kim, 1996	South Korea	RC	8	10	NR	NR	NR	NR	NR	NR	Basal ganglia	Basal ganglia	NR	NR	NR	NR	NR	NR	< 24	< 24
Cho, 2006	China	RCT	30	30	56.7 ± 8.7	56.6 ± 9.0	36.7	33.3	HTN (*n* = 23)	HTN (*n* = 22)	Deep Putamen (*n* = 21); Thalamus (*n* = 9)	Deep Putamen (*n* = 23) Thalamus (*n* = 7)	12	12	55.5 ± 23.3	32.2 ± 15.3	9.3 ± 1.2	10.1 ± 1.5	1.1	2.9
Nishihara, 2007	Japan	RC	27	20	65.3 ± 10.8	68.6 ± 11.6	29.6	50	NR	NR	Putamen (*n* = 17); Subcortical (*n* = 6); Cerebellum (*n* = 3); Thalamus (*n* = 1)	Putamen (*n* = 11); Thalamus (*n* = 3); Cerebellum (*n* = 3); Subcortical (*n* = 3)	NR	NR	50.5 ± 38.9	30.1 ± 25.2	NR	NR	39.4 ± 105.8	46.93 ± 143.40
Chi, 2014	China	RC	144	306	58.2 ± 17.5	57.3 ± 10.1	43.8	25.8	NR	NR	Deep (*n* = 71); Shallow (*n* = 41)	Deep (*n* = 79) Shallow (*n* = 227)	0	0	58.2 ± 17.5	61.5 ± 23.8	10.1 ± 1.7	11.4 ± 2.9	10.6 ± 9.8	15.7 ± 12.9
Cai, 2017	China	RC	20	22	59.6 ± 10.1	58.7 ± 12.4	45	40.9	NR	NR	Supratentorial intraparenchymal ICH	Supratentorial intraparenchymal ICH	NR	NR	51.7 ± 19.6	44.3 ± 18.0	7.6 ± 2.7	8.3 ± 2.0	NR	NR
Li, 2017	China	RC	32	36	60.7 ± 8.8	61.3 ± 8.4	56.3	55.6	HTN (*n* = 21); DM (*n* = 5)	HTN (*n* = 21); Diabetes (*n* = 9)	Lobar: Parietal (*n* = 15); Frontal (*n* = 3); Temporal (*n* = 9); Occipital (*n* = 5)	Lobar: Parietal (*n* = 13); Frontal (*n* = 6); Temporal (*n* = 10); Occipital (*n* = 7)	3	4	54.5 ± 14.2	53.8 ± 13.5	8.6 ± 3.0	8.6 ± 3.1	all < 24	all < 24
Li, 2018	China	RC	25	23	61.8 ± 6.7	62.5 ± 7.3	36	43.5	DM (*n* = 7); Obstructive hydrocephalus (*n* = 15)	DM (*n* = 7); Obstructive hydrocephalus (*n* = 15)	Cerebellum	Cerebellum	14	7	15.9 ± 3.5	15.5 ± 3.6	11.0 ± 3.0	11.6 ± 2.9	NR	NR
Dong, 2019	China	RC	39	42	50.5 ± 8.9	51.3 ± 8.0	30.8	42.9	NR	NR	Deep/basal ganglia (*n* = 26); Lobar/subcortical area (*n* = 13)	Deep/basal ganglia (*n* = 22) Lobar/subcortical area (*n* = 20)	NR	NR	56.8 ± 11.2	48.5 ± 11.5	8 ± 2.3	7 ± 2.3	28/39 < 6 h	27/42 < 6 h
Fu, 2019	China	RC	61	56	61.6 ± 9.2	65.6 ± 8.8	50.8	55.4	HTN (*n* = 48); DM (*n* = 14); Hyperlipidaemia (*n* = 11)	HTN (*n* = 42); DM (*n* = 11); Hyperlipidaemia (*n* = 10)	Deep/basal ganglia (*n* = 61)	Deep/basal ganglia (*n* = 56)	8	5	49.8 ± 11.3	45.4 ± 15.6	8.0 ± 2.9	8.4 ± 3.1	9.1 ± 3.1	10.2 ± 3.5
Guo, 2019	China	RC	27	40	59.8 ± 12.9	63.1 ± 12.7	48.1	32.5	HTN (*n* = 22)	HTN (*n* = 32)	Basal Ganglia (*n* = 27)	Basal Ganglia (*n* = 40)	17	22	50.4 ± 20.5	46.3 ± 17.7	8.2 ± 3.4	8.7 ± 2.8	5.2 ± 6.4	6.0 ± 5.8
Liu, 2020	China	RC	60	99	< 60 (*n* = 27) > 60 (*n* = 21)	< 60 (*n* = 44) > 60 (*n* = 55)	35	42.4	HTN (*n* = 51); DM (*n* = 5); Smoking (*n* = 22) Hx; CVD (*n* = 5)	HTN (*n* = 85); DM (*n* = 9); Smoking (*n* = 21); Hx; CVD (*n* = 13)	Deep/basal ganglia (*n* = 60)	Deep/basal ganglia (*n* = 99)	NR	NR	40–80 (*n* = 40) > 80 (*n* = 20)	40–80 (*n* = 68) > 80 (*n* = 31)	all < 8	all < 8	< 8 (*n* = 12) > 8 (*n* = 48)	< 8 (*n* = 34) > 8 (*n* = 65)
Mao, 2020	China	RC	63	54	56.5 ± 9.7	60.2 ± 11.9	25.4	35.2	NR	NR	Basal ganglia	Basal ganglia	0	0	47.8 ± 18.2	46.1 ± 18.2	9.0 (7.0–11.0)	10.0 (8.0–12.0)	NR	NR
Xiao, 2020	China	RC	30	78	61.3 ± 12.4	62.5 ± 12.3	46.7	48.7	HTN (*n* = 25)	HTN (*n* = 63)	Putamen (*n* = 19); Thalamus (*n* = 7); Subcortical (*n* = 4)	Putamen (*n* = 47); Thalamus (*n* = 18); Subcortical (*n* = 13)	6	17	43.1 ± 4.3	41.7 ± 4.4	8.2 ± 2.2	8.2 ± 2.3	6–24	6–24
Guo, 2020	China	RC	105	304	< 60 (*n* = 70) > 60 (*n* = 35)	< 60 (*n* = 178) > 60 (*n* = 126)	38.1	39.5	HTN (*n* = 88); Smoking (*n* = 31); CVD (*n* = 14); DM (*n* = 7)	HTN (*n* = 253); Smoking (*n* = 72); CVD (*n* = 37); DM (*n* = 26)	Basal Ganglia (*n* = 105)	Basal Ganglia (*n* = 304)	NR	NR	20–40 (*n* = 12) 40–80 (*n* = 48) > 80 (*n* = 45)	20–40 (*n* = 131) 40–80 (*n* = 134) > 80 (*n* = 39)	Drowsy (*n* = 12) Lethargic (*n* = 31) Light Coma (*n* = 53) Deep Coma (*n* = 9)	Drowsy (*n* = 65) Lethargic (*n* = 82) Light Coma (*n* = 122) Deep Coma (*n* = 35)	22.5	22.1
Li, 2021	China	RC	27	25	63.6 ± 10.9	65.7 ± 8.0	40.7	56	NR	NR	Cerebellum	Cerebellum	NR	NR	21.9 ± 7.2	21.6 ± 8.6	10.1 ± 2.6	9.0 ± 3.1	23.0 ± 23.8	17.2 ± 7.8
Du, 2021	China	RC	212	343	> 60 (*n* = 72)	> 60 (*n* = 134)	36.3	38.2	HTN (*n* = 175); Smoking (*n* = 95); CVD (*n* = 24); DM (*n* = 14)	HTN (*n* = 283); Smoking (*n* = 232); CVD (*n* = 42); DM (*n* = 27)	Basal Ganglia (*n* = 212)	Basal Ganglia (*n* = 343)	NR	NR	20–40 (*n* = 38) 40–80 (*n* = 134) > 80 (*n* = 40)	20–40 (*n* = 151) 40–80 (*n* = 149) > 80 (*n* = 43)	3–5 (*n* = 23) 6–8 (*n* = 98) 9–14 (*n* = 91)	3–5 (*n* = 61) 6–8 (*n* = 110) 9–14 (*n* = 172)	< 12 (*n* = 85) 12–24 (*n* = 96) > 24 (*n* = 31)	< 12 (*n* = 153) 12–24 (*n* = 128) > 24 (*n* = 62)
Cai, 2022	China	PC	40	42	57.7 ± 10.1	54.9 ± 10.5	40	21.4	NR	NR	NR	NR	NR	NR	43.7 ± 19.1	46.5 ± 20.1	9.1 ± 2.5	9.5 ± 2.3	NR	NR
Tahara, 2022	Japan	RC	3032	474	66.2 ± 13.4	65.6 ± 13.2	41.2	40.1	HTN (*n* = 2185); DM (*n* = 485); Hyperlipidemia (*n* = 263); CVD (*n* = 226)	HTN (*n* = 343); DM (*n* = 69); Hyperlipidemia (*n* = 42); CVD (*n* = 40)	Putamen (*n* = 1285); Thalamus (*n* = 530); Subcortical (*n* = 474); Cerebellum (*n* = 383); Brainstem (*n* = 6); Other (*n* = 61)	Putamen (*n* = 314); Subcortical (*n* = 61); Thalamus (*n* = 58); Cerebellum (*n* = 25); Brainstem (*n* = 0); Other (*n* = 7)	288	4	NR	NR	9.6 ± 3.7	10.4 ± 3.3	all < 48	all < 48
He, 2023	China	RC	64	58	54.0 ± 11.9	54.3 ± 10.4	29.7	25.9	HTN (*n* = 50); DM (*n* = 10); Hyperlipidaemia (*n* = 18)	HTN (*n* = 48); DM (*n* = 11); Hyperlipidaemia (*n* = 15)	Deep/basal ganglia (*n* = 64)	Deep/basal ganglia (*n* = 58)	18	14	47.7 ± 12.3	48.1 ± 12.3	8.1 ± 1.5	8.1 ± 1.6	10.3 ± 3.9	10.1 ± 3.6
Tan, 2023	China	RC	37	37	57.3 ± 11.1	57.3 ± 12.7	27	27	HTN (*n* = 32); DM (*n* = 11); CAD (*n* = 4); Prior stroke; (*n* = 20)	HTN (*n* = 33); DM (*n* = 9); CAD (*n* = 7); Prior stroke; (*n* = 20)	Supratentorial	Supratentorial	NR	NR	44.7 ± 10.9	44.5 ± 10.5	8.9 ± 0.5	8.9 ± 0.6	9.6 ± 9.6	40.4 ± 24.5
Song, 2023	China	RC	96	153	57.3 ± 10.7	58.0 ± 10.6	NR	NR	NR	NR	Supratentorial ICH (subcortex/basal ganglia/internal capsule/thalamus ± IVH)	Supratentorial ICH (subcortex/basal ganglia/internal capsule/thalamus ± IVH)	NR	NR	NR	NR	NR	NR	< 72	< 72
Jiang, 2024	China	RCT	47	44	58.1 ± 12.7	62.8 ± 10.8	29.8	36.4	HTN (*n* = 47); DM (*n* = 10); CAD (*n* = 1)	HTN (*n* = 44); DM (*n* = 13); CAD (*n* = 1)	Basal ganglia	Basal ganglia	21	13	40.3 ± 8.4	36.4 ± 5.5	8.6 ± 2.7	8.6 ± 2.1	6.3 ± 4.0	5.8 ± 3.1
Xie, 2024	China	RC	45	45	NR	NR	NR	NR	HTN (*n* = 45)	HTN (*n* = 45)	Supratentorial	Supratentorial	NR	NR	> 30	> 30	NR	NR	< 48	< 48
Yang, 2024	China	RC	36	39	66.3 ± 11.3	65.0 ± 12.1	27.8	33.3	Smoking (*n* = 12); Alcohol (*n* = 3); DM (*n* = 2); COPD (*n* = 21); CHD (*n* = 19);	Smoking (*n* = 20); Alcohol (*n* = 10); DM (*n* = 6); COPD (*n* = 19); CHD (*n* = 19);	Deep: left thalamus/internal capsule (*n* = 17)	Deep: left thalamus/internal capsule (*n* = 15)	19	9	24.2 ± 1.9	23.8 ± 3.4	10.0 ± 1.6	9.8 ± 1.4	4.0 (3.0–6.0)	4.0 (2.0–4.0)
Xu, 2024	China	RCT	239	246	56.6 ± 11.0	57.8 ± 11.0	30.5	30.9	DM (*n* = 19); CHD (*n* = 12); Prior Stroke (*n* = 20); Chronic Kidney (*n* = 6)	DM (*n* = 21); Ischemic Heart (*n* = 15); Prior Stroke (*n* = 18); Chronic Kidney (*n* = 8)	Basal Ganglia (*n* = 169); Lobar (*n* = 45); Thalamus (*n* = 25)	Basal Ganglia (*n* = 177); Lobar (*n* = 50); Thalamus (*n* = 19)	48	53	49.1 ± 20.3	48.5 ± 14.9	9.0 ± 3.1	9.4 ± 3.2	18 ± 14.1	19 ± 17.0
Murthy, 2025	USA	RC	391	312	60 ± 14.1	62 ± 16.3	42.6	46.1	HTN (*n* = 258); DM (*n* = 74); AF (*n* = 43); CHD (*n* = 35); Prior stroke/TIA (*n* = 70); Smoker (*n* = 50)	HTN (*n* = 220); DM (*n* = 69); AF (*n* = 38); CHD (*n* = 34); Prior stroke/TIA (*n* = 48); Smoker (*n* = 64)	Supratentorial	Supratentorial	NR	NR	NR	NR	NR	NR	2.0 ± 1.5	2.0 ± 1.5
Chen, 2025	China	RCT	19	20	69.0 ± 4.9	66.9 ± 5.1	31.6	45	Years of HTN Hx: 13.5 ± 1.97	Years of HTN Hx: 13.1 ± 2.42	Thalamic (*n* = 8); Basal Ganglia (*n* = 5); Lobar (*n* = 6)	Thalamic (*n* = 12); Basal Ganglia (*n* = 7); Lobar (*n* = 1)	NR (exclusion criteria)	NR (exclusion criteria)	47.2 ± 8.1	42.4 ± 6.8	7.5 ± 2.2	6.7 ± 1.9	< 24	< 24
Wu, 2025	China	RC	20	40	62.9 ± 11.36	59.8 ± 12.5	40	35	HTN 55%; DM 25%; CAD 5%	HTN 65%; DM 12.5%; CAD 7.5%	Basal ganglia	Basal ganglia	NR (massive IVH bleed excluded)	NR (massive IVH bleed excluded)	41.3 ± 11.9	40.6 ± 7.1	9.6 ± 1.8	9.5 ± 2.1	52.2 ± 14.6	51.3 ± 11.1
Guo, 2025	China	RC	65	182	58.3 ± 9.6	55.5 ± 11.4	30.8	36.8	HTN (*n* = 65); Smoking (*n* = 20); Alcohol (*n* = 17)	HTN (*n* = 182); Smoking (*n* = 52); Alcohol (*n* = 50)	Basal nuclei (*n* = 24); Frontal lobe (*n* = 24); Occipital (*n* = 17)	Basal nuclei (*n* = 72); Frontal lobe (*n* = 59); Occipital (*n* = 51)	NR	NR	51.6 ± 11.8	52.4 ± 13.6	9.2 ± 2.4	9.7 ± 3.1	< 48	< 48

Abbreviations: *ES* endoscopic surgery; *CA* catheter-based surgery; *RC* retrospective cohort study; *PC* prospective cohort study; *RCT* randomized control trial; *NR* not reported; *ICH* intracerebral hemorrhage; *IVH* intraventricular hemorrhage; *GCS* Glasgow coma scale; *HTN* hypertension; *DM* diabetes mellitus; *CVD* cardiovascular disease; *CHD* coronary heart disease; *TIA* transient ischemic attack

### Hematoma evacuation rate

**Fig. 2:**
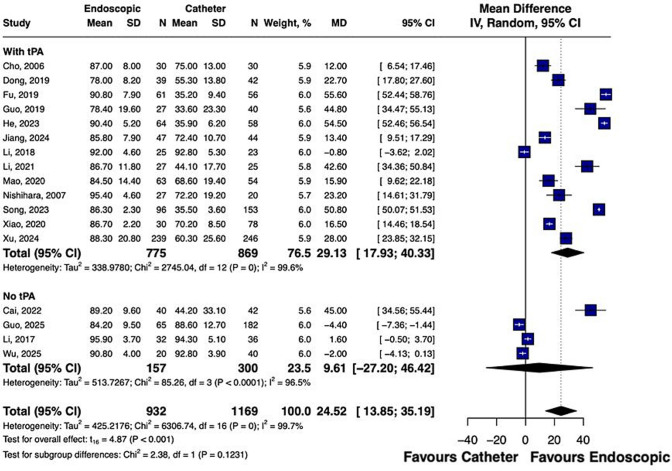
Hematoma evacuation rate on postoperative day one. Forest plot comparing hematoma evacuation rate on postoperative day one following endoscopic- or catheter-based minimally invasive surgery, with subgroup analysis by use of tissue plasminogen activator (tPA). Abbreviations: RR, risk ratio; IV, inverse variance; CI, confidence interval

Seventeen studies [4, 8, 10, 13, 15, 16, 20, 24, 31–33, 40, 45, 51, 57, 58, 60] (58.6%) reported postoperative day-one evacuation rate. Endoscopic cohorts had significantly higher evacuation rates than catheter-based cohorts (MD 24.52%; 95% CI: 13.85–35.19; *P* < 0.001; *I*^2^ = 99.7%) (Fig. 2). In subgroup analysis, endoscopy was also associated with higher evacuation rates than catheter-based approaches using tPA (MD 29.13; 95% CI: 17.93–40.33; *I*^2^ = 99.6%).

### Mortality

**Fig. 3:**
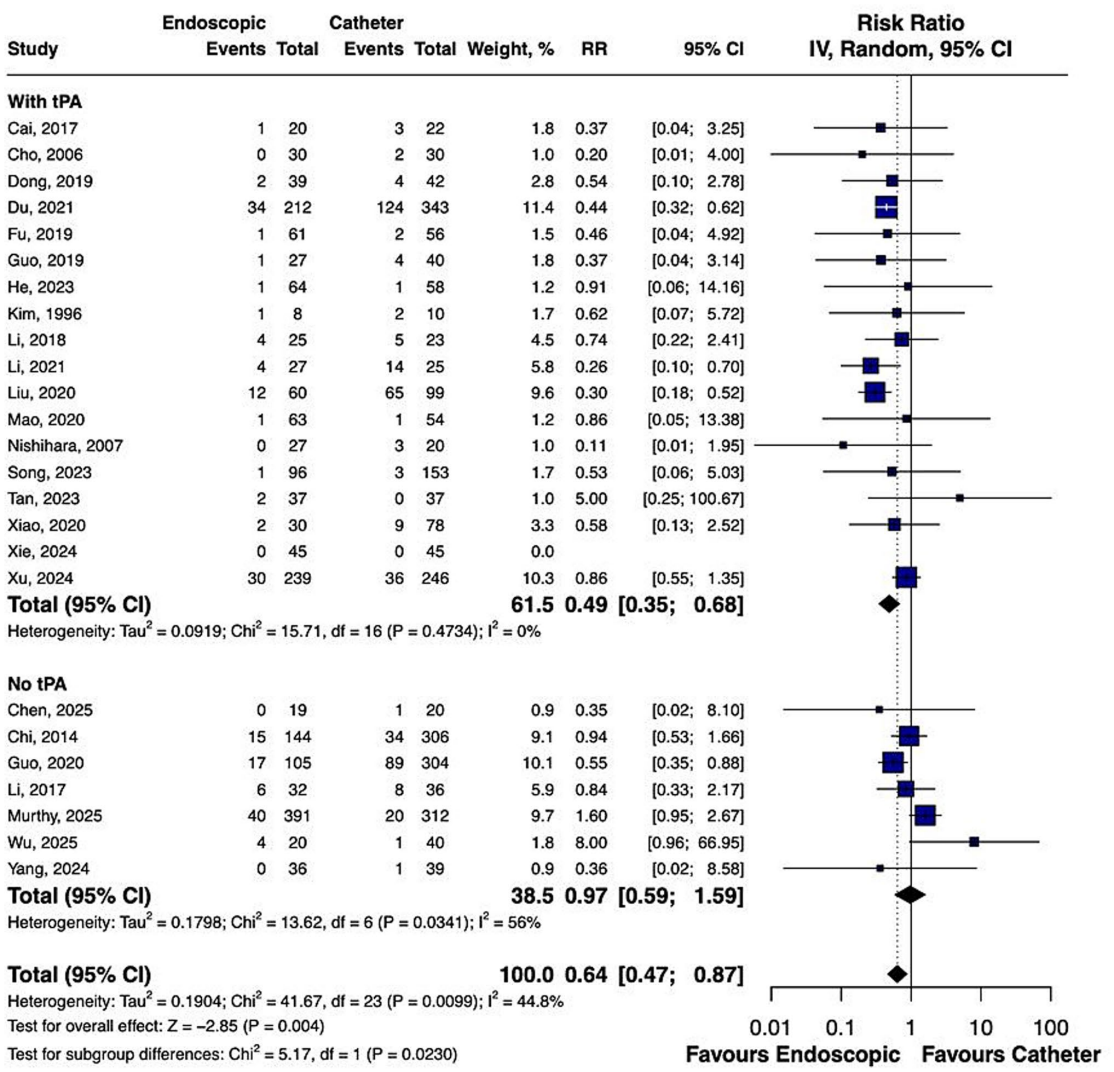
Mortality. Forest plot comparing mortality following endoscopic- or catheter-based minimally invasive surgery, with subgroup analysis by use of tissue plasminogen activator (tPA). Abbreviations: RR, risk ratio; IV, inverse variance; CI, confidence interval

Twenty-five studies [5–8, 10, 11, 13, 15, 17, 20, 28, 31–33, 35, 40, 44, 45, 51, 55, 57–61] (86.2%) reported mortality. Death occurred in 9.6% of endoscopic patients (179/1857) and 17.7% of catheter-based patients (432/2438), with a lower pooled risk with endoscopy (RR 0.64; 95% CI: 0.47–0.87; *P* < 0.01; *I*^2^ = 44.8%) (Fig. 3). On subgroup analysis, endoscopic cohorts had a significantly reduced risk compared to catheter-based with tPA cohorts (RR 0.49; 95% CI: 0.35–0.68; *I*^2^ = 0.0%), whereas no difference was observed versus catheter-based without tPA cohorts (RR 0.97; 95% CI: 0.59–1.59; *I*^2^ = 56.0%).

### Overall complications

Sixteen studies [5, 6, 8, 13, 16, 17, 20, 24, 28, 31–33, 40, 59–61] (55.2%) reported overall complications, occurring in 26.7% of endoscopic patients (216/808) and 25.9% of catheter-based patients (241/930), with no significant difference between strategies (RR 0.98; 95% CI: 0.78–1.25; *P* = 0.90; *I*^2^ = 48.5%) (Supplementary Fig. 1). Given the breadth of this composite and variable complication surveillance across studies, this finding reflects no detectable difference rather the demonstrated equivalence. The mechanism-specific safety endpoints (rebleeding and infection) are reported separately. In subgroup analyses overall complications were similar between endoscopy and catheter-based approaches both with and without tPA, with no significant difference between strata (test for subgroup difference *P* = 0.12).

### Rebleeding

Twenty-four studies [5, 6, 8, 10, 11, 13, 15–17, 20, 24, 28, 31–33, 35, 45, 51, 55, 57–61] (82.8%) reported rebleeding, which occurred in 4.7% of endoscopic patients (64/1371) and 8.5% of catheter-based patients (170/1992), favoring endoscopy (RR 0.58; 95% CI: 0.39–0.87; *P* < 0.01; *I*^2^ = 22.2%) (Supplementary Fig. 2). On subgroup analysis, endoscopic cohorts had a significantly reduced risk compared to catheter-based with tPA cohorts (RR 0.54; 95% CI: 0.33–0.88; *I*^2^ = 32.9%), whereas no significant difference was observed versus catheter-based without tPA cohorts (RR 0.75; 95% CI: 0.37–1.50; *I*^2^ = 0.0%).

### Infection

Twelve studies [5, 6, 8, 10, 16, 17, 24, 31, 40, 55, 57, 60] (41.4%) reported infection. Infection occurred in 10.3% of endoscopic patients (65/631) and 10.0% of catheter-based patients (78/780), with no significant difference (RR 1.03; 95% CI: 0.64–1.66; *P* = 0.05; *I*^2^ = 43.6%) (Supplementary Fig. 3).

### Secondary outcomes

Ten studies [4, 5, 8, 16, 31–33, 40, 45, 55, 61] (34.5%) reported postoperative residual hematoma volume; endoscopic cohorts had lower postoperative volumes than catheter-based cohorts (MD −5.6 mL; 95% CI: −10.30–0.90; *P* < 0.05; *I*^2^ = 96.9%) (Supplementary Fig. 4).

Four studies [13, 31–33] (13.8%) reported PHE, with no significant difference between strategies (Supplementary Fig. 5).

Eight studies [8, 10, 32, 33, 45, 55, 58, 60] (27.6%) reported ICU length of stay and ten studies [6, 8, 17, 20, 24, 33, 35, 55, 57, 60] (34.5%) reported hospital LOS, with no significant difference between cohorts (ICU: MD −1.5 days; 95% CI: −3.60–0.60; *P* = 0.16; *I*^2^ = 94.3%); Hospital: MD −0.2 days; 95% CI: −1.50–1.20; *P* = 0.79; *I*^2^ = 69.8%) (Supplementary Fig. 6–7).

Procedural metrics favored catheter-based approaches: operative time was shorter (seventeen studies [5, 6, 8, 10, 13, 17, 20, 24, 31, 33, 35, 40, 45, 51, 55, 57, 58, 60] (58.6%); MD 54.2 min; 95% CI: 38.20–70.10; *P* < 0.001; *I*^2^ = 98.3%) and intraoperative blood loss was lower (nine studies [6, 13, 16, 20, 24, 31, 33, 40, 60] (31.0%); MD 69.0 mL; 95% CI: 27.70–110.40; *P* < 0.001; *I*^2^ = 96.9%) (Supplementary Fig. 8–9).

Postoperative GCS/GOS favored endoscopy (SMD 0.27; 95% CI: 0.02–0.51; *P* < 0.05; *I*^2^ = 82.1%) (Supplementary Fig. 10), while mRS favored catheter-based cohorts (MD 0.28; 95% CI: 0.04–0.52; *P* < 0.05; *I*^2^ = 37.3%) (Supplementary Fig. 11). Leave-one-out sensitivity analysis for statistically significant outcomes did not materially change effect estimates.

### Sensitivity analysis

Baseline severity did not account for the safety or efficacy findings (Supplementary Table 5). For mortality, rebleeding, and evacuation rate, neither the between-arm difference in baseline hematoma volume nor in GCS significantly moderated the effect; where a borderline volume signal appeared (mortality), it favored greater endoscopic benefit in cohorts whose endoscopic arm was more severely affected. Randomized-trial estimates were concordant with observational cohorts (subgroup-difference *P* = 0.36, 0.46, and 0.31 for mortality, rebleeding, and evacuation rate), the evacuation advantage persisted among trials (MD 17.9%; 95% CI 7.8–27.9), and effects were directionally consistent across regions.

### Publication bias, risk of bias, and certainty of evidence

Funnel plots demonstrated no major asymmetry (Supplementary Fig. 12), and Egger’s regression did not indicate small-study effects (*P* = 0.09). Across included studies, 10 studies were judged at moderate risk of bias and 18 at serious risk, most commonly due to confounding and outcome measurement (Supplementary Table 6). Using GRADE, certainty of evidence for primary outcomes was rated as moderate, downgraded primarily for risk of bias (Supplementary Table 7). Previous systematic review addressing this topic were rated critically low quality by AMSTAR-2 (Supplementary Table 8).

## Discussion

This systematic review and meta-analysis synthesizes 29 comparative studies (8221 patients) evaluating two MIS strategies for supratentorial sICH: endoscopic and catheter-based evacuation (stereotactic aspiration or stereotactic drainage with or without adjunctive thrombolysis). In pooled analyses, endoscopic evacuation was associated with lower mortality, rebleeding, higher evacuation rate, and lower postoperative hematoma volume, while overall complications and infection were similar between approaches. Catheter-based techniques showed shorter operative time and lower intraoperative blood loss. These signals do not establish causality, but map onto biologically plausible mechanistic differences between approaches [9, 27].

These findings can be interpreted as different therapeutic kinetics. Endoscopic evacuation is an immediate, visually guided cavity intervention coupling mechanical debulking with targeted hemostasis under direct visualization [30]. Catheter-based approaches, by contrast, are trajectory-accurate but largely non-visual after placement, prioritizing minimal parenchymal transgression while achieving clearance via aspiration alone or staged drainage – sometimes augmented by enzymatic clot dissolution [21]. This distinction matters because the early post-ictus period is shaped by competing imperatives: rapid reduction of mass effect and toxic burden versus avoidance of renewed hemorrhage in a fragile vascular bed [36, 42].

### Hemostasis and early hazard: why mortality and rebleeding separate strategies

The most clinically consequential outcomes in these cohorts are mortality and rebleeding, clustering in a domain where the two strategies are fundamentally non-equivalent: hemostasis control in the immediate post-ictus window. Endoscopic evacuation couples clot removal to visual cavity management, including cavity inspection, and, where feasible, management of active bleeding points or diffuse oozing. Catheter-based approaches, by contrast, are largely non-visual after placement – hemostasis is therefore managed indirectly via systemic parameters and time, rather than by cavity-level control [23].

This distinction becomes particularly relevant when thrombolysis is used. The subgroup structure is notable: the reduction in mortality and rebleeding with endoscopy was most pronounced in comparisons against catheter drainage with thrombolysis, whereas comparisons against catheter strategies without thrombolysis are neutral. This does not prove thrombolysis causality, but it aligns with a coherent biological framework: thrombolysis perturbs clot stability in tissue already vulnerable to microvascular injury, blood–brain barrier disruption, and ongoing hypertensive stress[18, 19, 34]. Even when thrombolysis improves drainage kinetics, it can widen the window for secondary hemorrhage if cavity level hemostasis is not secured or if catheter position is suboptimal [43, 49]. Endoscopy’s capability to couple evacuation with immediate cavity assessment provides one plausible explanation for why rebleeding and mortality differences manifest most clearly in the thrombolysis subgroup.

### Evacuation kinetics and secondary injury biology: volume, edema, and what “clearance” actually means

The hematoma is not inert volume but is simultaneously a space-occupying lesion and a biomechanical source of secondary injury: thrombin signaling, erythrocyte lysis, iron-mediated toxicity, oxidative stress, microglial activation, and inflammatory amplification in the perihematomal region [56]. A strategy that more reliably reduces residual hematoma volume may therefore attenuate both mechanical and molecular components of injury, although direct causal linkage requires prospective data with standardized endpoints [12].

Two findings illustrate how evacuation is not a single construct. Postoperative hematoma volume and day-one hematoma evacuation rate favored endoscopic in the pooled analysis. However, evacuation metrics are definition dependent: scan timing, baseline volume definition, and thrombolysis use materially affect percentage-based measures [38]. Conversely, endoscopy may reduce residual volume more definitively even when “day-one” percentage metrics are noisy or inconsistently measured.

PHE is a particularly instructive endpoint because it is jointly driven by hematoma biology and iatrogenic factors, as well as contributing to elevation of ICP and neurological deterioration [34, 48]. In this review, PHE outcomes were sparse and did not demonstrate a difference. That absence should not be overread as it likely reflects limited reporting and heterogeneous measurement rather than true equivalence [42]. Future work would benefit from standardized PHE measurement timepoints and volumetric definitions [9, 48].

### Procedural efficiency, complications, and the problem of composite endpoints

The procedural efficiency findings are consistent and clinically relevant: catheter-based strategies were associated with shorter operative time and lower blood loss. In sICH populations enriched for advanced age, hypertensive vasculopathy, and antithrombotic exposure, these “operative physiology” endpoints are non-trivial [21]. They likely reflect the workflow simplicity of catheter-based approaches: smaller access corridors, less tissue manipulation, and minimal cavity-level handling.

However, efficiency does not guarantee neutrality on safety endpoints dependent on hemostasis biology and postoperative trajectory. In this review, overall complications and infection were similar between strategies. That finding is reassuring but should be treated as no detectable difference rather than definitive equivalence, because “overall complications” is a composite outcome prone to definitional drift, variable surveillance, and selective reporting across cohorts [22]. Infection risk likewise depends strongly on perioperative protocols and, in catheter-based strategies, the duration of indwelling devices and ICU practices – factors inconsistently captured in most datasets [37]. The practical implication is that complication neutrality does not contradict the possibility of mechanism-specific differences in select adverse events, particularly where subgroup signals are present.

Functional outcomes remain the most difficult domain to interpret. Endoscopy was associated with a modest improvement in early neurological scores (GCS/GOS), whereas catheter-based cohorts showed slightly better mRS in fewer studies. This divergence is plausible without invoking contradiction: these scales sample different constructs, and mRS is especially sensitive to baseline severity, hematoma location, rehabilitation access, and survivorship effects. Moreover, the pooled mRS difference (0.28 points) lies well below the ~ 1-pooint change generally regarded as clinically meaningful, limiting its clinical relevance even where statistically significant. Where mortality differs, disability metrics can also shift due to differential survival of more severely affected patients. Robust inference requires standardized follow-up timepoints and covariate-adjusted analyses that are rarely available in the current evidence base [1].

### Selecting technique by hemostatic and kinetic demands

Clinically, this review supports a phenotype-contingent framing rather than a binary preference. Endoscopic evacuation may be best aligned to scenarios in which immediate cavity control and hemostatic certainty are prioritized, or hematoma phenotypes where direct visualization and mechanical debulking achieve meaningful residual volume reduction without prolonged reliance on lysis kinetics [46]. Catheter-based aspiration without thrombolysis may be best aligned to patients in whom procedural physiological cost must be minimized and where clot characteristics and access geometry predict adequate clearance without enzymatic destabilization [23]. Catheter drainage with thrombolysis may be valuable when staged reduction is acceptable and protocol fidelity is high but should be approached as a distinct biological intervention rather than a generic efficiency upgrade [21].

### Limitations and methodological considerations

Several constraints warrant cautious interpretation. Most included studies were observational, and risk of bias was frequently moderate-to-serious, particularly from confounding and outcome measurement. Notably, the endoscopic arm presented with larger baseline hematomas and lower preoperative GCS – given that both predict worse outcomes, this imbalance would be expected to attenuate rather than inflate the observed endoscopic benefit, an interpretation supported by meta-regression and randomized trial-restricted analyses. Residual confounding from unmeasured factors – including center-level technical preference and hemostasis thresholds – nonetheless cannot be excluded. The evidence base was geographically concentrated in East Asia, and predominantly China (25/29 studies), which may limit generalizability to European, North American, and other health systems, given that regional differences in perioperative management, rehabilitation pathways, device availability, and surgical technique could influence the magnitude of the observed associations. In the region-stratified sensitivity analysis, the mortality association did not differ significantly between Chinese and non-Chine cohorts (RR 0.58 versus 0.81; between-region *P* = 0.64), although data from outside Chine was sparse. Technique labels were heterogeneous, while key modifiers such as patient age, hemorrhage pattern, intraventricular extension, and timing-to-intervention were not uniformly reported, despite their recognized influence on outcomes after intracranial hemorrhage [39]. Several continuous outcomes showed substantial heterogeneity, consistent with variability in imaging timepoints and outcome definitions [48]. Finally, functional outcomes were inconsistently reported and temporally heterogeneous, limiting linkage between early physiological effects and long-term disability.

### Future directions

Future work should shift from broad “MIS versus MIS” comparisons toward phenotype-stratified, technique-specific evaluation. Priorities include prospective head-to-head trials or registries comparing endoscopic evacuation, catheter-based evacuation without thrombolysis, and catheter-based evacuation with thrombolysis within prespecified hematoma phenotypes. Studies should adopt standardized imaging schedules and harmonized definitions for evacuation rate, postoperative residual volume, rebleeding, and PHE [48]. Safety reporting should be prespecify and prioritize discrete, hemostasis-linked endpoints – symptomatic rebleeding, procedure-related infection, and complications requiring intervention – rather than a single broad composite. Reporting should also incorporate fidelity metrics, including catheter placement accuracy, completeness of endoscopic evacuation, and thrombolysis dosing and dwell protocols. Finally, outcomes should be patient-centered, separating early neurological recovery from longer-term disability, and accounting for survivorship effects and goals-of-care decisions.

## Conclusion

In this comparative synthesis of MIS strategies for supratentorial sICH, endoscopic evacuation was associated with lower mortality, rebleeding, postoperative hematoma volume, and higher evacuation rates, with comparable overall complications and infection. Catheter-based strategies had shorter operative time and lower intraoperative blood loss. Given predominantly non-randomized evidence, these are strategy-specific associations; subgroup patterns by thrombolysis support treating catheter evacuation with and without thrombolysis as distinct interventions. Head-to-head trials or well-adjusted prospective registries should standardize evacuation and edema metrics, prespecify hemostasis-linked safety endpoints, and report functional outcomes at fixed timepoints.

## Supplementary Information

Below is the link to the electronic supplementary material.

Supplementary file1 (DOCX 3902 KB)

## Data Availability

No datasets were generated or analysed during the current study.

## Abbreviations

CA: Catheter Aspiration
CI: Confidence Intervals
GCS: Glasgow Coma Scale
GOS: Glasgow Outcome Score
ICU: Intensive Care Unit
LOS: Length of Stay
MD: Mean Difference
MIS: Minimally Invasive Surgery
mRS: Modified Rankin Scale
PHE: Perihematomal Edema
RCT: Randomized Controlled Trial
ROBINS-I: Risk of Bias in Non-Randomised Studies of—Interventions
RR: Risk Ratio
sICH: Spontaneous Intracerebral Hemorrhage
SMD: Standardized Mean Difference
