# Supplementary material for: Endoscopic versus catheter-based minimally invasive evacuation of spontaneous supratentorial intracerebral hemorrhage: a systematic review and meta-analysis
Source: Neurosurg Rev. 2026 Aug 5;49(1):512. doi: 10.1007/s10143-026-04426-3 (PMC13437680; doi:10.1007/s10143-026-04426-3)
Supplement: Supplementary file 1 [file 10143_2026_4426_MOESM1_ESM.docx]

| **Database** | **Search Strategy** |
| --- | --- |
| PubMed  (n=461) | ("Intracranial Hemorrhages"[Mesh] OR "Cerebral Hemorrhage"[Mesh] OR "Hemorrhagic Stroke"[Mesh] OR "Intracranial Hemorrhage, Hypertensive"[Mesh] OR "Basal Ganglia Hemorrhage"[Mesh] OR "Putaminal Hemorrhage"[Mesh] OR encephalorrhagia[tiab] OR hematencephalon[tiab] OR ICH[tiab] OR ICHs[tiab] OR IPH[tiab] OR IPHs[tiab])  AND  ("endoscopic surgery"[tiab] OR "neuroendoscopic surgery"[tiab] OR "endoscopic evacuation"[tiab] OR "neuroendoscopic evacuation"[tiab] OR "minimal invasive surgery"[tiab] OR "minimally invasive surgery"[tiab] OR endoscop*[tiab] OR neuroendoscop*[tiab] OR "neuroendoscopy"[mh:noexp] OR "Apollo"[tiab] OR “Artemis"[tiab]) OR "Nagasaka"[tiab] OR "SCUBA"[tiab] OR "mixed fluid"[tiab] OR "dry field"[tiab] OR "air method"[tiab] OR "air-only"[tiab])  AND  ("Drainage"[Mesh] OR "Thrombolytic Therapy"[Mesh] OR "Stereotaxic Techniques"[Mesh] OR catheter*[tiab] OR cannula*[tiab] OR stereotac*[tiab] OR stereotax*[tiab] OR aspiration[tiab] OR thromboly*[tiab] OR fibrinoly*[tiab] OR "alteplase"[tiab] OR "rtPA"[tiab] OR "urokinase"[tiab] OR "MISTIE"[tiab] OR "ADIWIT"[tiab] OR "CRANIO-CAN"[tiab] OR "soft channel"[tiab] OR "hard channel"[tiab] |
| Embase  (n=776) | (exp "Intracranial Hemorrhages"/ OR exp "Cerebral Hemorrhage"/ OR exp "Hemorrhagic Stroke"/ OR exp "Intracranial Hemorrhage, Hypertensive"/ OR exp "Basal Ganglia Hemorrhage"/ OR exp "Putaminal Hemorrhage"/ OR encephalorrhagia.tw. OR hematencephalon.tw. OR ICH.tw. OR ICHs.tw. OR IPH.tw. OR IPHs.tw.)  AND  ("endoscopic surgery".tw. OR "neuroendoscopic surgery".tw. OR "endoscopic evacuation".tw. OR "neuroendoscopic evacuation".tw. OR "minimal invasive surgery".tw. OR "minimally invasive surgery".tw. OR endoscop*.tw. OR neuroendoscop*.tw. OR neuroendoscopy/ OR Apollo.tw. OR Artemis.tw.) OR Nagasaka.tw. OR SCUBA.tw. OR "mixed fluid".tw. OR "dry field".tw. OR "air method".tw. OR air-only.tw.)  AND  (exp Drainage/ OR exp "Thrombolytic Therapy"/ OR exp "Stereotaxic Techniques"/ OR catheter*.tw. OR cannula*.tw. OR stereotac*.tw. OR stereotax*.tw. OR aspiration.tw. OR thromboly*.tw. OR fibrinoly*.tw. OR alteplase.tw. OR rtPA.tw. OR urokinase.tw. OR MISTIE.tw. OR ADIWIT.tw. OR CRANIO-CAN.tw. OR "soft channel".tw. OR "hard channel".tw.) |
| CENTRAL  (n=103) | ([mh "Intracranial Hemorrhages"] OR [mh "Cerebral Hemorrhage"] OR [mh "Hemorrhagic Stroke"] OR [mh "Intracranial Hemorrhage, Hypertensive"] OR [mh "Basal Ganglia Hemorrhage"] OR [mh "Putaminal Hemorrhage"] OR encephalorrhagia:ti,ab OR hematencephalon:ti,ab OR ICH:ti,ab OR ICHs:ti,ab OR IPH:ti,ab OR IPHs:ti,ab)  AND  ("endoscopic surgery":ti,ab OR "neuroendoscopic surgery":ti,ab OR "endoscopic evacuation":ti,ab OR "neuroendoscopic evacuation":ti,ab OR "minimal invasive surgery":ti,ab OR "minimally invasive surgery":ti,ab OR endoscop*:ti,ab OR neuroendoscop*:ti,ab OR [mh ^neuroendoscopy] OR Apollo:ti,ab OR Artemis:ti,ab) OR Nagasaka:ti,ab OR SCUBA:ti,ab OR "mixed fluid":ti,ab OR "dry field":ti,ab OR "air method":ti,ab OR air-only:ti,ab)  AND  ([mh Drainage] OR [mh "Thrombolytic Therapy"] OR [mh "Stereotaxic Techniques"] OR catheter*:ti,ab OR cannula*:ti,ab OR stereotac*:ti,ab OR stereotax*:ti,ab OR aspiration:ti,ab OR thromboly*:ti,ab OR fibrinoly*:ti,ab OR alteplase:ti,ab OR rtPA:ti,ab OR urokinase:ti,ab OR MISTIE:ti,ab OR ADIWIT:ti,ab OR CRANIO-CAN:ti,ab OR "soft channel":ti,ab OR "hard channel":ti,ab) |

**Supplementary Table 1*:* Search Strategies**

Full electronic search strategies used to identify relevant studies for this review, reported for each database. n = number of articles returned.

| **Author, Year** | **Intervention description** | | **Operative time (mins)** | | **Procedure-related complications** | | **Systemic complications** | | **Follow-up (months)** | |
| --- | --- | --- | --- | --- | --- | --- | --- | --- | --- | --- |
|  | **ES** | **CA** | **ES** | **CA** | **ES** | **CA** | **ES** | **CA** | **ES** | **CA** |
| **Cho, 2006**^1^ | Burrhole + 4-mm Karl Storz endoscope with irrigation system | Stereotactic aspiration (Archimedean screw aspirator + silicon catheter). Urokinase given. | 158.1 ± 47.4 | 117.4 ± 23.3 | 0 | CNS infection (n=1) | 0 | 0 | 6 | 6 |
| **Dong, 2019**^2^ | Burrhole + 4-mm Karl Storz endoscope with irrigation system | CT guided minimally invasive aspiration: puncture and drainage. Urokinase given. | 170.0 ± 28.0 | 55.0 ± 16.1 | Intracranial infection (n=1); wound infection (n=1) | Intracranial infection (n=2) | Pneumonia (n=1) | Pneumonia (n=2) | 3 | 3 |
| **Chi, 2014**^3^ | Neuroendoscopic keyhole surgery | Stereotactic drilling with directional catheter drainage | NR | NR | NR | NR | NR | NR | 3–6 | 3–6 |
| **Fu, 2019**^4^ | Craniotomy (2.5-3cm), then transparent sheath + endoscope introduced into spave | Soft catheter into hematoma through a burr hole, then aspirated with 5ml syringe. Urokinase given. | 87.2 ± 28.9 | 38.1 ± 18.6 | Meningitis (n=2) | Meningitis (n=5) | Pulmonary infection (n=3); GI bleed (n=3); epilepsy (n=1) | Pulmonary infection (n=4); GI bleed (n=2); epilepsy (n=2) | 6 | 6 |
| **He, 2023**^5^ | Bone window (2-2.5cm) then 3.6mm neuroendoscope (Karl Storz) | Minimally invasive puncture and drainage (1cm hole), aspiration with 5ml syringe then urokinase | 78.7 ± 22.5 | 39.4 ± 20.3 | Meningitis (n=1) | Meningitis (n=2) | Pulmonary infection (n=3); GI bleed (n=3); epilepsy (n=1) | Pulmonary infection (n=5); GI bleed (n=3); epilepsy (n=2) | 6 | 6 |
| **Liu, 2020**^6^ | Endoscopic evaucation via 3-5cm boneflap, puncturing cannula positioned in centre of haematoma then clot aspiration with 5ml syringe | Stereotactic aspiration: burrhole (1cm) and soft catheter inserted. Aspirated with 5ml syringe. Urokinase given. | NR | NR | NR | NR | NR | NR | 6 | 6 |
| **Wu, 2025**^7^ | Endoscopic evacuation via 3 cm bone window | Robot-assisted stereotactic drilling + catheter aspiration | 221.1 ± 60.1 | 152.7 ± 38.5 | Intracranial infection (n=1); rebleeding (n=0) | Intracranial infection (n=1); rebleeding (n=0) | Pulmonary infection (n=6); UTI (n=2); ventilation >96h (n=5) | Pulmonary infection (n=5); UTI (n=1); ventilation >96h (n=6) | NR | NR |
| **Mao, 2020**^8^ | Keyhole (small craniotomy) minimally invasive neuroendoscopic hematoma evacuation | Stereotactic catheter insertion + repeated urokinase clot lysis + drainage | 68.4 ± 36.7 | 11.7 ± 4.1 | Intracranial infection (n=0) | Intracranial infection (n=1) | Pneumonia (n=4);  UTI (n=13);  DVT (n=5) | Pneumonia (n=2);  UTI (n=7);  DVT (n=4) | 6 | 6 |
| **Jiang, 2024**^9^ | Neuronavigation-assisted neuroendoscopic hematoma removal | Neuronavigation-assisted intracerebral hematoma puncture & drainage ± urokinase | 118.0 ± 33.3 | 39.7 ± 13.1 | Intracranial infection (n=3);  rebleeding (n=2);  seizure (n=1) | Intracranial infection (n=11); rebleeding (n=5);  seizure (n=1) | Pulmonary infection (n=8); DVT (n=1) | Pulmonary infection (n=10); DVT (n=0) | 1 | 1 |
| **Tan, 2023**^10^ | Neuro-endoscopic hematoma evacuation | Robot-assisted stereotactic hematoma drainage ± urokinase | 163.2 ± 39.6 | 47.4 ± 23.4 | Intracranial infection (n=3); rebleeding (n=2) | Intracranial infection (n=0); rebleeding (n=1) | 0 | 0 | 3–12 | 3–12 |
| **Kim, 1996**^11^ | Stereotactic endoscopic removal | Stereotactic catheter drainage + urokinase | NR | NR | Rebleeding (n=1) | 0 | Cardiac arrest (n=1) | Cardiac arrest (n=1); pulmonary edema (n=2) | 6 | 6 |
| **Li, 2021**^12^ | Endoscopic evacuation | Catheter aspiration + urokinase | NR | NR | 0 | Rebleeding (n=2) | Pulmonary infection (n=2) | 0 | 6 | 6 |
| **Li, 2018**^13^ | Endoscopic surgery | Stereotactic aspiration + urokinase | 129.4 ± 15.1 | 58.8 ± 6.2 | Infectious meningitis (n=1) | Infectious meningitis (n=1) | Pulmonary infection (n=3); hypoproteinemia (n=5); digestive tract ulcer (n=4); renal failure (n=1) | Pulmonary infection (n=2); hypoproteinemia (n=2); digestive tract ulcer (n=2); renal failure (n=1) | 12 | 12 |
| **Song, 2023**^14^ | Neuroendoscopic hematoma evacuation (STORZ endoscope) | Burr hole stereotactic drilling + catheter drainage + urokinase | 69.5 ± 9.3 | 47.3 ± 9.7 | NR | NR | NR | NR | 3–6 | 3–6 |
| **Cai, 2017**^15^ | Endoscopic surgery | Catheter-based surgery (burr hole + urokinase + drainage) | 68.3 ± 8.4 | 36.9 ± 7.1 | 0 | Intracranial infection (n=3) | Pneumonia (n=1) | Pneumonia (n=2) | NR | NR |
| **Cai, 2022**^16^ | Neuroendoscopic hematoma evacuation | Stereotactic aspiration | NR | NR | NR | NR | NR | NR | NR | NR |
| **Xie, 2024**^17^ | Neuroendoscopy: 1cm bone hole drilled into skull, puncture cannula inserted to establish surgical channel, haematoma removed with endoscopic assistance | Stereotactic puncture surgery: Puncture needle introduced to aspirate half of haematoma. Then haematoma infused with urokinase then fine silicone drainage tube inserted | NR | NR | NR | NR | NR | NR | NR | NR |
| **Chen, 2025**^18^ | Neuroendoscopic: bone flap drilled, tube core lens set was used to access haematoma cavity. tube core removed and rigid observation lens inserted to aspirate haematoma from multiple angles | Minimally invasive soft channel puncture and drainage: burrhole drilled in skull, silicone tube (F12-14) inserted, YL-1 puncture needle used to aspirate haematoma with 10ml syringe. | 141.3 ± 14.9 | 79.9 ± 16.3 | 0 | Intracranial infection (n=2) | Pulmonary infection (n=3); stress ulcer (n=3) | Pulmonary infection (n=4); stress ulcer (n=3); renal failure (n=2) | 3 | 3 |
| **Yang, 2024**^19^ | 2-3cm bony window created, transparent sheath layer put into haematoma cavity to remove haematoma, endoscope introduced + haematoma cleared | Stereotactic surgery with puncture at the centre of the haematoma | NR | NR | NR | NR | Pneumonia (n = 13); GI bleeding (n = 11); DVT (n = 4) | Pneumonia (n = 10); GI bleeding (n = 6); DVT (n = 4) | 6 | 6 |
| **Murthy, 2025**^20^ | Endoscopic surgery | Stereotactic drainage | NR | NR | NR | NR | NR | NR | NR | NR |
| **Guo, 2025**^21^ | 2-3cm bone flap, endoscopic drill sheath advanced to haematoma site. sheath served as endoscopic guide and placed at angles to clear haematoma. | 8mm hole created through scalp and skull, puncture needle placed at centre of haematoma, drainage tube connected and needle core withdrawn. syringe used to aspirate. | NR | NR | Intracranial infection (n=5) | Intracranial infection (n=8) | Pneumonia (n=4); renal insufficiency (n=3) | Pneumonia (n=9); renal insufficiency (n=8) | 6 | 6 |
| **Nishihara, 2007**^22^ | Burrhole followed by endoscopic sheath inserted through burrhole | Stereotactic guided burrhole. 3mm aspiration needle used to evacuate hamatoma with syringe. Urokinase given. | 71.7 ± 22.5 | 101.9 ± 18.1 | NR | NR | NR | NR | 6 | 6 |
| **Guo, 2020**^23^ | Endoscopic aspiration | Stereotactic aspiration | NR | NR |  |  | NR | NR | NR | NR |
| **Xu, 2024**^24^ | 2cm bone flap followed by transparent working channel to evacuate haematoma under endoscopic surveillance | Stereotactic aspiration: catheter insertion followed by aspiration. Urokinase injected through catheter. | 120 ± 42 min | 60 ± 42 min | Intracranial infection (n=11) | Intracranial infection (n=17) | Pneumonia (n=54) | Pneumonia (n=45) | 6 | 6 |
| **Xiao, 2020**^25^ | 3cm bone window. Puncture made and transparent working sheath left, serving as a conduit for endoscopic procedure: endoscope inserted and aspirator suctioning at various degrees. | Puncture and drainage. CT located haematoma, blood withdrawn with a syringe. Urokinase given. | 82.4 ± 5.7 | 40.1 ± 11.5 | Meningitis (n=2) | Meningitis (n=18) | Pulmonary infection (n=27); epilepsy (n=5); GI bleeding (n=4) | Pulmonary infection (n=75); epilepsy (n=14); GI bleeding (n=8) | 3 | 3 |
| **Du, 2021**^26^ | 3cm bone flap, transparent sheath inserted via puncturing cannula, clot aspirated with syringe | Stereotactic aspiration. Burrhole drilled, soft catheter inserted, clot pumped with syringe. Urokinase injected. | NR | NR | NR | NR | NR | NR | 6 | 6 |
| **Li, 2017**^27^ | 2cm burrhole, transparent plastic sheath of 10mm diameter inserted into haematoma. 4mm rod-lens endoscope with irrigation system (Karl Storz) introduced into sheath. Haematoma evacuated with aspirator. | Stereotactic aspiration. 10-French cannula into centre of haematoma using stereotactic frame + aspiration. | 126.1 ± 16.5 | 64.7 ± 8.6 | Epilepsy (n=5); meningitis (n=1) | Epilepsy (n=4) meningitis (n=3) | Pneumonia (n=3); GI ulcer (n=7); renal failure (n=1) | Pneumonia (n=3); GI ulcer (n=5); renal failure (n=1) | 6 | 6 |
| **Tahara, 2022**^28^ | Endoscopic Evacuation | Stereotactic aspiration | NR | NR | NR | NR | NR | NR | NR | NR |
| **Guo, 2019**^29^ | Neuroendoscopy: 2-3cm craniotomy made, neuro-endoscopic puncture and working channel system used to create operating space for neuroendoscope. Neuroendoscope advanced through transparent sheath. haematoma evacuation via suction catheter. | Burrhole drainage: burrhole then drainage connected to a three-way stopcock placed via burrhole. Haematoma aspirated through syringe connected to drainage. Urokinase administered before removal of drainage. | 79.4 ± 9.2 | 37.3 ± 8.7 | Intracranial infection (n=2) | Intracranial infection (n=1) | GI bleeding (n=3); pneumonia (n=1) | Pneumonia (n=3); GI bleeding (n=2) | NR | NR |

**Supplementary Table 2: Surgical characteristics of included studies.**

Abbreviations: ES, endoscopic surgery; CA, catheter-based surgery; NR, not reported; CT, computed tomography

| **Variable** | **Endoscopic Surgery** | **Catheter-based surgery** | **Mean difference (95% Confidence Interval)** | **P-value** |
| --- | --- | --- | --- | --- |
| Hematoma volume (mL) | 45.04 | 41.43 | 1.81 (0.40 – 3.23) | *P* < 0.05 |
| Pre-operative GCS | 8.94 | 9.13 | -0.28 (-0.55 – -0.01) | *P* < 0.05 |
| Time to surgery (hours) | 15.8 | 18.9 | -2.45 (-7.19 – 2.30) | *P* = 0.31 |

**Supplementary Table 3: Baseline hematoma volume, pre-operative GCS, and time to surgery in endoscopic vs catheter-based surgery groups.**

Abbreviations: GCS, Glasgow Coma Scale

| **No.** | **Item** | **Outcome** |
| --- | --- | --- |
| 1 | Did the research questions and inclusion criteria for the review include the components of PICO? | Y |
| 2 | *Did the report of the review contain an explicit statement that the review methods were established prior to the conduct of the review and did the report justify any significant deviations from the protocol? | Y |
| 3 | Did the review authors explain their selection of the study designs for inclusion in the review? | Y |
| 4 | *Did the review authors use a comprehensive literature search strategy? | Y |
| 5 | Did the review authors perform study selection in duplicate? | Y |
| 6 | Did the review authors perform data extraction in duplicate? | Y |
| 7 | *Did the review authors provide a list of excluded studies and justify the  exclusions? | Y |
| 8 | Did the review authors describe the included studies in adequate detail? | Y |
| 9 | *Did the review authors use a satisfactory technique for assessing the  risk of bias (RoB) in individual studies that were included in the review? | Y |
| 10 | Did the review authors report on the sources of funding for the studies  included in the review? | Y |
| 11 | *If meta-analysis was performed did the review authors use appropriate  methods for statistical combination of results? | Y |
| 12 | If meta-analysis was performed, did the review authors assess the potential impact of RoB in individual studies on the results of the meta-analysis or other evidence synthesis? | Y |
| 13 | *Did the review authors account for RoB in individual studies when interpreting/ discussing the results of the review? | Y |
| 14 | Did the review authors provide a satisfactory explanation for, and discussion of, any heterogeneity observed in the results of the review? | Y |
| 15 | *If they performed quantitative synthesis did the review authors carry out an adequate investigation of publication bias (small study bias) and discuss its likely impact on the results of the review? | Y |
| 16 | Did the review authors report any potential sources of conflict of interest, including any funding they received for conducting the review? | Y |

**Supplementary Table 4: AMSTAR-2 quality appraisal**

AMSTAR-2 criteria applied to the present review, with item-level judgements (Y = yes; PY = partial yes; N = no). *= Critical weaknesses

| **Analysis** | **k** | **Effect (95% CI)** | **I², %** | **P-value** |
| --- | --- | --- | --- | --- |
| **Mortality — risk ratio (RR<1 favors endoscopy)** | | | | |
| Overall | 24 | 0.64 (0.47, 0.87) | 44.8 | 0.004† |
| Catheter-based with tPA | 17 | 0.49 (0.35, 0.68) | 0.0 |  |
| Catheter-based without tPA | 7 | 0.97 (0.59, 1.59) | 56.0 | 0.023‡ |
| Randomized trials | 3 | 0.82 (0.53, 1.27) | 0.0 |  |
| Observational cohorts | 21 | 0.63 (0.45, 0.88) | 48.4 | 0.355‡ |
| China | 21 | 0.58 (0.44, 0.77) | 22.9 |  |
| Outside China | 3 | 0.81 (0.21, 3.21) | 46.8 | 0.636‡ |
| *Meta-regression: baseline volume (per mL)* | 18 | 0.93 (0.87, 1.00) | — | 0.055 |
| *Meta-regression: baseline GCS (per point)* | 17 | 0.71 (0.48, 1.06) | — | 0.097 |
| **Rebleeding — risk ratio (RR<1 favors endoscopy)** | | | | |
| Overall | 23 | 0.58 (0.39, 0.87) | 22.1 | 0.008† |
| Catheter-based with tPA | 18 | 0.54 (0.33, 0.88) | 32.9 |  |
| Catheter-based without tPA | 5 | 0.75 (0.37, 1.50) | 0.0 | 0.446‡ |
| Randomized trials | 4 | 0.79 (0.32, 1.97) | 16.1 |  |
| Observational cohorts | 19 | 0.54 (0.34, 0.84) | 24.4 | 0.463‡ |
| China | 21 | 0.57 (0.38, 0.86) | 25.0 |  |
| Outside China | 2 | 0.98 (0.07, 13.60) | 30.0 | 0.690‡ |
| *Meta-regression: baseline volume (per mL)* | 17 | 0.96 (0.88, 1.06) | — | 0.436 |
| *Meta-regression: baseline GCS (per point)* | 16 | 0.80 (0.30, 2.14) | — | 0.662 |
| **Day-1 evacuation rate — mean difference, % (positive favors endoscopy)** | | | | |
| Overall | 17 | 24.52 (14.63, 34.42) | 99.7 | <0.001† |
| Catheter-based with tPA | 13 | 29.13 (19.01, 39.25) | 99.6 |  |
| Catheter-based without tPA | 4 | 9.61 (−12.77, 32.00) | 96.5 | 0.119‡ |
| Randomized trials | 3 | 17.87 (7.81, 27.93) | 93.8 |  |
| Observational cohorts | 14 | 26.00 (14.18, 37.81) | 99.8 | 0.305‡ |
| China | 16 | 24.61 (14.10, 35.12) | 99.8 |  |
| Outside China | 1 | 23.20 (−118.45, 164.85) | 0.0 | 0.984‡ |
| *Meta-regression: baseline volume (per mL)* | 16 | −0.19 (−1.63, 1.25) | — | 0.793 |
| *Meta-regression: baseline GCS (per point)* | 15 | 5.85 (−13.38, 25.08) | — | 0.551 |

**Supplementary Table 5: Sensitivity analyses for the efficacy outcomes**

†Test for overall effect. ‡Test for subgroup differences (Cochran Q). GCS, Glasgow Coma Scale; tPA, tissue plasminogen activator.

**Supplementary Table 6A: Risk of bias assessment (non-randomised)**

Summary of risk of bias assessment for non-randomised studies (ROBINS-I), reported by individual domain and overall risk of bias.

**Supplementary Table 6B: Risk of bias assessment (randomised)**

Summary of risk of bias assessment for randomised studies (RoB 2), reported by individual domain and overall risk of bias.

**Supplementary Table 7: Certainty of evidence (GRADE)**

GRADE assessment of certainty of evidence for each primary outcome in the present review.

| **Author** | **Critical Flaws** | **Non-Critical Flaws** | **Overall Confidence** |
| --- | --- | --- | --- |
| Yang et al. (2024) | **Item 7** - No list of excluded studies provided  **Item 9** - Inappropriate risk of bias assessment performed - used Newcastle-Ottawa scale  **Item 13** - Risk of bias not considered in results | **Item 10** - Funding sources of included studies not reported  **Item 12** - Did not explore impact of RoB | **Critically Low** |
| Sun et al. (2024) | **Item 2** - No explicit protocol registered before conducting the review  **Item 7** - No list of excluded studies provided  **Item 9** - Inappropriate risk of bias assessment performed - used Newcastle-Ottawa scale | **Item 10** - Funding sources of included studies not reported  **Item 12** - Did not explore impact of RoB | **Critically Low** |
| Wang et al. (2024) | **Item 2** - No explicit protocol registered before conducting the review  **Item 7** - No list of excluded studies provided  **Item 9** - Inappropriate risk of bias assessment performed - used Newcastle-Ottawa scale  **Item 13** - Risk of bias not considered in results | **Item 10** - Funding sources of included studies not reported  **Item 12** - Did not explore impact of RoB | **Critically Low** |
| Patel et al., 2025 (this review) | **None** | **None** | **High** |

**Supplementary Table 8: AMSTAR-2 quality appraisal of prior reviews**

Summary of AMSTAR-2 assessment of previously published systematic reviews on this topic, indicating critical and non-critical flaws, and overall confidence rating.

**Supplementary Figure 1: Overall complications**

Forest plot comparing overall complications following endoscopic- or catheter-based minimally invasive surgery, with subgroup analysis by use of tissue plasminogen activator (tPA). Abbreviations: RR, risk ratio; IV, inverse variance; CI, confidence interval

**Supplementary Figure 2: Rebleeding**

Forest plot comparing rebleeding following endoscopic- or catheter-based minimally invasive surgery, with subgroup analysis by use of tissue plasminogen activator (tPA). Abbreviations: RR, risk ratio; IV, inverse variance; CI, confidence interval

**Supplementary Figure 3: Infection**

Forest plot comparing infection following endoscopic- or catheter-based minimally invasive surgery. Abbreviations: RR, risk ratio; IV, inverse variance; CI, confidence interval

**Supplementary Figure 4: Postoperative hematoma volume**

Forest plot comparing postoperative hematoma volume following endoscopic- or catheter-based minimally invasive surgery. Abbreviations: MD, mean difference; IV, inverse variance; CI, confidence interval

**Supplementary Figure 5: Perihematomal edema**

Forest plot comparing volume of perihematomal edema following endoscopic- or catheter-based minimally invasive surgery. Abbreviations: MD, mean difference; IV, inverse variance; CI, confidence interval

**Supplementary Figure 6: ICU length of stay**

Forest plot comparing intensive care unit (ICU) length of stay following endoscopic- or catheter-based minimally invasive surgery. Abbreviations: MD, mean difference; IV, inverse variance; CI, confidence interval

**Supplementary Figure 7: Hospital length of stay**

Forest plot comparing hospital length of stay (LOS) following endoscopic- or catheter-based minimally invasive surgery. Abbreviations: MD, mean difference; IV, inverse variance; CI, confidence interval

**Supplementary Figure 8: Operative time**

Forest plot comparing operative time following endoscopic- or catheter-based minimally invasive surgery. Abbreviations: MD, mean difference; IV, inverse variance; CI, confidence interval

**Supplementary Figure 9: Intraoperative blood loss**

Forest plot comparing intraoperative blood loss following endoscopic- or catheter-based minimally invasive surgery. Abbreviations: MD, mean difference; IV, inverse variance; CI, confidence interval

**Supplementary Figure 10: Postoperative GCS/GOS**

Forest plot comparing Glasgow Coma Scale (GCS) or Glasgow Outcome Scale (GOS) following endoscopic- or catheter-based minimally invasive surgery. Abbreviations: MD, mean difference; IV, inverse variance; CI, confidence interval

**Supplementary Figure 11: mRS**

Forest plot comparing modified Rankin Scale (mRS) following endoscopic- or catheter-based minimally invasive surgery. Abbreviations: MD, mean difference; IV, inverse variance; CI, confidence interval

**Supplementary Figure 12: Publication bias assessment**

Funnel plot of effect estimates from included studies plotted against their respective standard errors for overall complications.

**Reference list of included studies**

Cho DY, Chen CC, Chang CS, Lee WY, Tso M. Endoscopic surgery for spontaneous basal ganglia hemorrhage: comparing endoscopic surgery, stereotactic aspiration, and craniotomy in noncomatose patients. *Surg Neurol*2006;65(6):547-55. doi:10.1016/j.surneu.2005.09.032

Dong J, Jiang Z, Chen J, Huang N, Chen J, Liu G. Evacuation assisted by endoscopy has better efficacy for supratentorial intracerebral hematoma than CT-guided minimally invasive aspiration: a retrospective observational cohort study. *Br J Neurosurg* 2019;33(3):348-51. doi:10.1080/02688697.2018.1559273

Chi FL, Lang TC, Sun SJ, et al. Relationship between different surgical methods, hemorrhage position, hemorrhage volume, surgical timing, and treatment outcome of hypertensive intracerebral hemorrhage. *World J Emerg Med* 2014;5(3):203-8. doi:10.5847/wjem.j.issn.1920-8642.2014.03.008

Fu C, Wang N, Chen B, et al. Surgical management of moderate basal ganglia intracerebral hemorrhage: comparison of safety and efficacy of endoscopic surgery, minimally invasive puncture and drainage, and craniotomy. *World Neurosurg* 2019;122:e995-e1001. doi:10.1016/j.wneu.2018.10.192

He H, Wang F, Bao D, Niu C. Comparison of endoscopic evacuation, craniotomy, and puncture aspiration for the treatment of spontaneous basal ganglia intracerebral hematoma. *Clin Neurol Neurosurg* 2023;233:107957. doi:10.1016/j.clineuro.2023.107957

Liu H, Wu X, Tan Z, et al. Long-term effect of endoscopic evacuation for large basal ganglia hemorrhage with GCS scores ≤8. *Front Neurol* 2020;11:848. doi:10.3389/fneur.2020.00848

Wu H, Lu B, Wang W, et al. Efficacy and prognosis of rosa robot-assisted stereotactic intracranial hematoma removal in patients with cerebral hemorrhage in basal ganglia region: comparison with craniotomy and neuroendoscopy. *Transl Stroke Res* 2025;16(5):1594-605. doi:10.1007/s12975-025-01330-8

Mao JL, Xu YG, Luo YC, et al. Keyhole approach endoscopic surgery versus stereotactic aspiration plus urokinase in treating basal ganglia hypertensive intracerebral hemorrhage [in Chinese]. *Zhongguo Yi Xue Ke Xue Yuan Xue Bao* 2020;42(4):513-20. doi:10.3881/j.issn.1000-503X.12591

Jiang L, Tian J, Guo C, et al. Comparison of the efficacy of neuronavigation-assisted intracerebral hematoma puncture and drainage with neuroendoscopic hematoma removal in treatment of hypertensive cerebral hemorrhage. *BMC Surg* 2024;24(1):86. doi:10.1186/s12893-024-02378-3

Tan K, Peng Y, Li J, Liu C, Tao L. Long-term outcomes and cost-effectiveness evaluation of robot-assisted stereotactic hematoma drainage for spontaneous intracerebral hemorrhage. *Front Neurol* 2023;14:1291634. doi:10.3389/fneur.2023.1291634

Kim MH, Song JH, Kim SH, Park DB, Shin KM. A new trend in operative technique for intracerebral hemorrhage: a comparative study of stereotactic endoscopic removal and stereotactic catheter drainage. *Neurosurg Focus* 1996;1(4):e2. doi:10.3171/foc.1996.1.4.2

Li L, Liu H, Luo J, et al. Comparison of long-term outcomes of endoscopic and minimally invasive catheter evacuation for the treatment of spontaneous cerebellar hemorrhage. *Transl Stroke Res* 2021;12(1):57-64. doi:10.1007/s12975-020-00827-8

Li L, Li Z, Li Y, et al. Surgical evacuation of spontaneous cerebellar hemorrhage: comparison of safety and efficacy of suboccipital craniotomy, stereotactic aspiration, and thrombolysis and endoscopic surgery. *World Neurosurg* 2018;117:e90-e98. doi:10.1016/j.wneu.2018.05.170

Song P, Lei P, Li Z, et al. Post-operative rebleeding in patients with spontaneous supratentorial intracerebral hemorrhage: factors and clinical outcomes. *Am J Transl Res* 2023;15(8):5168-83.

Cai Q, Zhang H, Zhao D, et al. Analysis of three surgical treatments for spontaneous supratentorial intracerebral hemorrhage. *Medicine (Baltimore)* 2017;96(43):e8435. doi:10.1097/MD.0000000000008435

Cai Q, Wang W, Li Z, et al. New approach of minimally invasive evacuation for spontaneous supratentorial intracerebral hemorrhage. *Am J Transl Res* 2022;14(3):1969-78.

Xie Q, Zhang X, Ding Y, Zhang L. Assessing the clinical efficacy of stereotactic puncture in combination with postoperative rehabilitation training for hypertensive cerebral hemorrhage. *Altern Ther Health Med*2024;30(6):122-7.

Chen S, Feng Y, Chen C. Evaluation of three minimally invasive surgical techniques for hematoma removal in elderly patients with hypertensive intracerebral hemorrhage: a pilot randomized study. *Neurol Asia*2025;30(2):403-10. doi:10.54029/2025xzt

Yang S, Liu Y, Wang S, et al. Stereotactic puncture surgery combined with early hyperbaric oxygen therapy may be better for long-term functional recovery in patients with moderate amounts of thalamic-inner capsule region haemorrhage. *Clin Neurol Neurosurg* 2024;241:108287. doi:10.1016/j.clineuro.2024.108287

Murthy SB, Zhang C, Garton ALA, et al. Outcomes following minimally invasive surgery for intracerebral hemorrhage in the AHA Get With The Guidelines-Stroke Registry. *Stroke* 2025;56(6):1441-50. doi:10.1161/STROKEAHA.124.048650

Guo T, Zhu Q, Zhu C, et al. Comparative analysis of neurological function and prognosis after stereotactic aspiration and neuroendoscopic surgery for hypertensive intracerebral hemorrhage. *Ann Ital Chir*2025;96(4):514-22. doi:10.62713/aic.3950

Nishihara T, Morita A, Teraoka A, Kirino T. Endoscopy-guided removal of spontaneous intracerebral hemorrhage: comparison with computer tomography-guided stereotactic evacuation. *Childs Nerv Syst*2007;23(6):677-83. doi:10.1007/s00381-007-0325-6

Guo W, Liu H, Tan Z, et al. Comparison of endoscopic evacuation, stereotactic aspiration, and craniotomy for treatment of basal ganglia hemorrhage. *J Neurointerv Surg* 2020;12(1):55-61. doi:10.1136/neurintsurg-2019-014962

Xu X, Zhang H, Zhang J, et al. Minimally invasive surgeries for spontaneous hypertensive intracerebral hemorrhage (MISICH): a multicenter randomized controlled trial. *BMC Med* 2024;22(1):244. doi:10.1186/s12916-024-03468-y

Xiao T, Wan J, Qu H, Jiang W, Zhou X. Endoscopic surgery versus minimal puncture drainage surgery for treatment of supratentorial intracerebral hemorrhage. *Turk Neurosurg* 2020;30(4):565-72. doi:10.5137/1019-5149.JTN.27977-19.2

Du Y, Gao Y, Liu HX, et al. Long-term outcome of stereotactic aspiration, endoscopic evacuation, and open craniotomy for the treatment of spontaneous basal ganglia hemorrhage: a propensity score study of 703 cases. *Ann Transl Med* 2021;9(16):1289. doi:10.21037/atm-21-1612

Li Y, Yang R, Li Z, et al. Surgical evacuation of spontaneous supratentorial lobar intracerebral hemorrhage: comparison of safety and efficacy of stereotactic aspiration, endoscopic surgery, and craniotomy. *World Neurosurg* 2017;105:332-40. doi:10.1016/j.wneu.2017.05.134

Tahara S, Hattori Y, Aso S, et al. Outcomes after endoscopic evacuation versus evacuation using craniotomy or stereotactic aspiration for spontaneous intracerebral hemorrhage: analysis using a Japanese nationwide database. *Neurocrit Care* 2023;38(3):667-75. doi:10.1007/s12028-022-01634-9

29.	Guo AS, Lin GS, Xie DH, et al. Effectiveness of surgical treatments for basal ganglia hemorrhage and imaging factors affecting hematoma evacuation rate by neuroendoscopic surgery. *J Neurol Surg A Cent Eur Neurosurg*2020;81(2):155-62. doi:10.1055/s-0039-1698523
